# Hearing loss in inherited peripheral neuropathies: Molecular diagnosis by NGS in a French series

**DOI:** 10.1002/mgg3.839

**Published:** 2019-08-08

**Authors:** Justine Lerat, Corinne Magdelaine, Anne‐Françoise Roux, Léa Darnaud, Hélène Beauvais‐Dzugan, Steven Naud, Laurence Richard, Paco Derouault, Karima Ghorab, Laurent Magy, Jean‐Michel Vallat, Pascal Cintas, Eric Bieth, Marie‐Christine Arne‐Bes, Cyril Goizet, Caroline Espil‐Taris, Hubert Journel, Annick Toutain, Jon Andoni Urtizberea, Odile Boespflug‐Tanguy, Fanny Laffargue, Philippe Corcia, Laurent Pasquier, Mélanie Fradin, Sylva Napuri, Jonathan Ciron, Jean‐Marc Boulesteix, Franck Sturtz, Anne‐Sophie Lia

**Affiliations:** ^1^ University of Limoges, MMNP Limoges France; ^2^ Service Oto‐Rhino‐Laryngologie et Chirurgie Cervico‐Faciale CHU Limoges Limoges France; ^3^ Service Biochimie et Génétique Moléculaire CHU Limoges Limoges France; ^4^ Laboratoire de Génétique Moléculaire CHU Montpellier Montpellier France; ^5^ University of Montpellier Montpellier France; ^6^ CRMR Neuropathies Périphériques Rares, CHU Limoges Limoges France; ^7^ Service de Neurologie et d'explorations fonctionnelles CHU Toulouse Toulouse France; ^8^ Service de Neurologie, Centre de référence de pathologie neuromusculaire CHU Toulouse Toulouse France; ^9^ Service de Génétique Médicale CHU Toulouse Toulouse France; ^10^ Service de Neurogénétique CHU Bordeaux Bordeaux France; ^11^ Service de Génétique médicale CHU Bordeaux Bordeaux France; ^12^ Service de Génétique Médicale CH Bretagne Atlantique Vannes France; ^13^ Service de Génétique CHU Tours Tours France; ^14^ Centre de référence Neuromusculaire Hôpital marin Hendaye France; ^15^ Service de Neurogénétique Hôpital Robert‐Debré AP‐HP Paris France; ^16^ Service de Génétique médicale CHU Clermont‐Ferrand Clermont‐Ferrand France; ^17^ Service de Neurologie CHU Tours Tours France; ^18^ Service de Génétique médicale CHU Rennes Rennes France; ^19^ Service de Pédiatrie CHU Rennes Rennes France; ^20^ Service de Neurologie CHU Poitiers Poitiers France; ^21^ Service Neurologie CHU Cahors Cahors France

**Keywords:** Charcot‐Marie‐Tooth, hearing loss, neuropathy, NGS

## Abstract

**Background:**

The most common inherited peripheral neuropathy is Charcot‐Marie‐Tooth disease (CMT), with a prevalence of 1/2500. Other symptoms can be associated to the condition, such as hearing loss. Currently, no global hearing impairment assessment has been determined, and the physiopathology is not well known.

**Methods:**

The aim of the study was to analyze among a French series of 3,412 patients with inherited peripheral neuropathy (IPN), the ones who also suffer from hearing loss, to establish phenotype‐genotype correlations. An NGS strategy for IPN one side and nonsyndromic hearing loss (NSHL) on the other side, were performed.

**Results:**

Hearing loss (HL) was present in only 44 patients (1.30%). The clinical data of 27 patients were usable. Demyelinating neuropathy was diagnosed in 15 cases and axonal neuropathy in 12 cases. HL varied from mild to profound. Five cases of auditory neuropathy were noticed. Diagnosis was made for 60% of these patients. Seven novel pathogenic variants were discovered in five different genes: *PRPS1; MPZ*; *SH3TC2*; *NEFL;* and *ABHD12*. Two patients with *PMP22* variant, had also an additional variant in *COCH* and *MYH14* respectively. No pathogenic variant was found at the DFNB1 locus. Genotype‐phenotype correlations do exist, especially with *SH3TC2, PRPS1, ABHD12, NEFL, a*nd *TRPV4.*

**Conclusion:**

Involvement of *PMP22* is not enough to explain hearing loss in patients suffering from IPN. HL can be due to cochlear impairment and/or auditory nerve dysfunction. HL is certainly underdiagnosed, and should be evaluated in every patient suffering from IPN.

## 1 INTRODUCTION

The most common inherited peripheral neuropathy is Charcot‐Marie‐Tooth disease (CMT), with a prevalence of 1/2500. CMT has a wide range of phenotypes and is genetically heterogeneous. *PMP22* duplication was the first identified pathogenic variant in 1992, and accounts for 15% of CMT patients (Timmerman et al., 1992). More than 90 genes are involved in the different types, which can be demyelinating, axonal, or intermediate with variable inheritance and expression. Other symptoms can be associated to the condition, such as scoliosis or hearing loss. Currently, no global hearing impairment assessment has been determined, and the physiopathology is not well‐known. The hypothesis of retrocochlear dysfunction has been suggested (Anzalone, Nuhanovic, Olund, & Carlson, 2018). It is therefore supposed that profound hearing impairment could be the result of cochlear nerve desynchronization, leading to auditory neuropathy.

Almost 10% of the French population suffers from hearing loss, which can be sensorineural, conductive, or mixed. Sensorineural hearing loss can be due to a virus (e.g. CytoMegaloVirus), environment (e.g. noise exposure), or genetic factors. Congenital hearing loss represents more than 50% of sensorineural hearing loss. More than 100 genes have been identified to be responsible for NSHL, and more still for syndromic hearing loss. Genetic hearing loss is most of the time a monogenic disease.

The aim of the study was to analyze a French series of patients suffering from inherited peripheral neuropathy associated with hearing loss, in order to establish phenotype‐genotype correlations.

## 2 MATERIALS AND METHODS

### 2.1 Patients

A French series of 3,412 patients suffering from IPN has been analysed thanks to medical records and a clinical questionnaire, so as to identify patients presenting IPN and hearing loss. The 3,412 patients had been selected on clinical and inheritance criterion, and all have been genetically screened.

Phenotypes were screened on the basis of clinical data and electroneuromyograms (ENMG) for IPN, and audiograms, OtoAcoustic Emissions (OAE), and Auditory Brainstem Responses (ABR) for hearing loss.

Peripheral blood samples of the patients were collected on EDTA tubes after giving their informed consent. The protocol was in accordance with French ethical legislation and Helsinki declaration.

### 2.2 Pathogenic variant detection

Genomic DNA was extracted by standard methods (Illustra DNA Extraction kit BACC3, GEHC). For neuropathy screening, a Next Generation Sequencing (NGS) strategy was implemented using a 92‐gene custom panel designed for CMT and associated neuropathies diagnosis (Table S1). It included the 44 known CMT genes, 27 genes involved in HSN (Hereditary Sensitive Neuropathy) and HMN (Hereditary Motor Neuropathy) and 21 other genes of interest involved in neuropathies of differential diagnosis. The amplified library was prepared with Ion P1 HiQ Template OT2 200 kit (Ampliseq Custom [Life technologies]), sequenced on Proton sequencer (Life technologies), and mapped to the human reference sequence GHCh37. Variants were assessed with Alamut Mutation Interpretation Software (Interactive Biosoftware, Rouen, France). Databases such as ExAC Genome browser (http://exac.broadinstitute.org), dbSPN135 (National Center for Biotechnology Information [NCBI], Bethesda, Maryland, USA, http://www.ncbi.nlm.nih.gov/projects/SPN/), ClinVar (www.ncbi.nlm.nih.gov/clinvar), and HGMD (www.hgmd.cf.ac.uk) were also screened. In silico studies were performed thanks to Polyphen‐2 (http://genetics.bwh.harvard.edu/pph2/), SIFT (https://sift.bii.a-star.edu.sg), UMD‐Predictor (http://umd-predictor.eu/), and Mutation Taster (http://www.mutationtaster.org/). Pathogenic variants of interest were verified by Sanger sequencing using forward and reverse primer pairs. Data have been submitted into a freely accessible public database, namely LOVD at https://databases.lovd.nl/shared/genes/DMD.

For hearing loss screening, MLPA and Sanger sequencing for *GJB2* and *GJB6* were performed for all the deaf patients. A NGS strategy was performed on a 63‐gene custom panel designed for hearing loss in 8 selected patients (Baux et al., 2017) (Table S2). Variants of interest were verified by Sanger sequencing using forward and reverse primer pairs.

Literature analysis has identified 36 genes described to be involved in both IPN and hearing loss (Table 1). These 36 genes are all present in the 92‐gene custom panel designed for CMT and IPN.

**Table 1:** The 36 genes of interest involved in both IPN and HL and their reference sequence

*Genes described to be involved in NP + HL*							
*AARS*	NM_001605.2	*DNAJB2*	NM_006736.5	*PEX12*	NM_000286.2	*SH3TC2*	NM_024577.3
*ABHD12*	NM_015600.4	*INF2*	NM_022489.3	*PEX7*	NM_000288.3	*SLC5A7*	NM_021815.2
*AIFM1*	NM_004208.3	*KIF5A*	NM_004984.2	*PHYH*	NM_001323082.1	*SLC25A46*	NM_138773.2
*DNMT1*	NM_001130823.1	*MFN2*	NM_014874.3	*PMP22*	NM_000304.2	*SOX10*	NM_006941.3
*FIG4*	NM_014845.5	*MPZ*	NM_001315491.1	*POLG*	NM_001126131.1	*SPTLC1*	NM_001281303.1
*GBE1*	NM_000158.3	*MYH14*	NM_001145809.1	*PRPS1*	NM_002764.3	*SURF1*	NM_003172.3
*GJB1*	NM_000166.5	*NDRG1*	NM_006096.3	*SBF2*	NM_030962.3	*TRPV4*	NM_021625.4
*GJB3*	NM_001005752.1	*NEFL*	NM_006158.3	*SCN9A*	NM_002977.3	*TTR*	NM_000371.3
*GLA*	NM_000169.2	*PDK3*	NM_001142386.2	*SETX*	NM_015046.5	*TYMP*	NM_001113755.1

## 3 RESULTS

### 3.1 Clinical description

Among the series of 3,412 patients, we had the information in medical records and clinical questionnaire of hearing loss associated with IPN in only 44 patients (1.30%). The clinical data of 27 patients, 15 women and 12 men, were usable for this study. The clinical description is presented in Table 2. The mean age was 60.77 years (from 10 to 90).

**Table 2:** Phenotypes of our 27 patients

Patient			Polyneuropathy				Hearing loss		Other symptoms
Reference Family	Patient (gender/age in years)	Form (Fam/spo)/ AD, AR or X‐linked	Neuropathy	Pes cavus	VCM (m/s)	Age at onset (years)	Degree	Age at onset (years)	
I	F, 87	Spo/ NA	Sensori‐motor Demyelinating	Y	48	12	NC	NC	Urinary incontinence, Small legs
II	F, 90	Spo/ NA	Sensori‐motor Axonal	Y	NC	61	Severe AN	68	/
III	M, 80	Fam/ AD	Sensori‐motor Axonal	Y	56	59	Moderate	NC	Cataracts, Retinal detachment
IV	M, 35	Spo/ NA	Sensori‐motor Axonal	*N*	20–30	8	Profound	1	Optic Neuropathy, Balance disorder
V	F, 86	Fam/AD	Sensori‐motor Demyelinating	Y	18	68	Progressive	NC	Balance disorder
VI	M, 88	Fam/AD	Sensori‐motor Demyelinating	Y	31	72	NC	NC	Ataxia, Alzheimer disease
VII	F, 60	Fam/AD	Demyelinating	NC	NC	NC	Moderate	NC	Balance disorder
VIII	F, 47	Spo/NA	Sensori‐motor Demyelinating	Y	27	4	NC	NC	Optic Neuropathy
IX	F, 47	Fam/AD	Sensori‐motor Axonal	Y	NC	44	Moderate	35	Pain, Chronic Respiratory Insufficiency
X	M, 69	Fam/AD	Sensori‐motor Demyelinating	NC	NC	NC	NC	NC	Severe form
X1	F, 68	Fam/AD	Sensori‐motor Axonal	Y	43	35	Moderate and progressive	35	/
XII	M, 33	Fam/AD	Sensori‐motor Demyelinating	Y	36	20	Severe AN	5	Tomacular neuropathy
XIII	F, 34	Spo/ NA	Sensori‐motor Demyelinating	Y	NC	12	Profound	1	Pain, Primary amenorrhea, oesophagus atresia
XIV	F, 68	Fam/AD	Sensori‐motor Demyelinating	NC	30	50	Severe to Profound AN	50	Balance disorders, Cochlear Implantation
XV	M, 29	Spo/NA	Sensori‐motor Axonal	Y	58	< 5	NC	< 5	/
XVI	F, 75	Spo/NA	Sensori‐motor Demyelinating	Y	51	< 5	NC	NC	/
XVII	F, 68	Spo/NA	Sensori‐motor Demyelinating	Y	34–37	9	Moderate	60	Scoliosis
XVIII	M, 68	Fam/AD	Sensori‐motor Axonal	Y	NC	65	Moderate AN	62	Bilateral Vocal cord Paresis
XIX	F, 69	Fam/AD	Sensori‐motor Axonal	Y	NC	45	Moderate	45	
XX	M, 83	Fam/AR	Sensori‐motor Demyelinating	Y	31	73	Moderate	60	
XXI	F, 10	Fam/AD	Sensori‐motor Demyelinating	Y	NC	2	Moderate	1	/
XXII	F, 80	Spo/ NA	Sensori‐motor Axonal	Y	NC	45	NC	NC	Scoliosis, Cataracts
XXIII	M, 19	Spo/ NA	Sensori‐motor Axonal	Y	52	11	Mild	6	Urinary incontinence, wheelchair
XXIV	F, 71	Spo/ NA	Sensori‐motor Demyelinating	Y	41	45	NC	< 5	Scoliosis
XXV	M, 78	Fam/AD	Sensori‐motor Axonal	Y	46	NC	NC	NC	Ataxia, Gougerot‐Sjogren
XXVI	M, 38	Spo/ NA	Sensori‐motor Demyelinating	NC	25–30	15	Moderate to profound AN	5	Cataracts, Ataxia
XXVII	M, 61	Spo/ NA	Sensori‐motor Axonal	Y	44	NC	Fluctuating	54	/

Abbreviations: AN, auditory neuropathy; F, female; Fam, familial; M, male; NA, not Available; NC, not communicated; Spo, sporadic.

Demyelinating neuropathy was diagnosed in 15 cases and axonal neuropathy in 12 cases. Age at onset varied from 2 to 73 years. In case of early onset, both demyelinating and axonal forms were observed (Patients I, IV, VIII, XIII, XV, XVI, XVII, XXI, XXIII and XXVI).

Hearing loss varied from mild to profound, could be progressive, and five cases of auditory neuropathy (AN) were related (Patients II, XII, XIV, XVIII and XXVI). Endocochlear involvement was also present with absence of Otoacoustic Emissions as in case of patient XIV. Age at onset varied from 1 to 68. One patient has successful cochlear implantation (Patient XIV) and one patient has recently been assessed for a cochlear implant (Patient XII). They both suffered from AN. In case of early onset (*n* = 8 cases), HL was severe to profound in four cases (Patients IV, XII, XIII and XXVI).

IPN and HL can occur nearly simultaneously in some patients, as in the case with patients XIV or patient XIX*,* or closely as in patients IV, XI, XVIII, XXI or XXVI. In contrast, the two features occured with a delay of up to 40 years, in others as in patients XXIV, and also patients XII, XVII and XX. Most of the time, hearing loss preceded IPN by several decades.

The other major symptoms observed were linked to other cranial nerve disorder such as optic neuropathy (*n* = 2), or bilateral laryngeal nerve paresis (*n* = 1). Neurological features such as cerebellar ataxia (*n* = 3), proprioceptive balance disorders (*n* = 4), urinary incontinence (*n* = 2) and pain (*n* = 2) were observed. Ophthalmological conditions like cataracts (*n* = 3) or retinal detachment (*n* = 1) were also present. Scoliosis was present in three cases. 2.

### 3.2 Genetic testing

Thirteen sporadic and 14 familial cases were noted. Inheritance mode was in favour of an autosomal dominant (AD) way in 13 cases, and an autosomal recessive (AR) one in 1 case.

**Figure 1:**
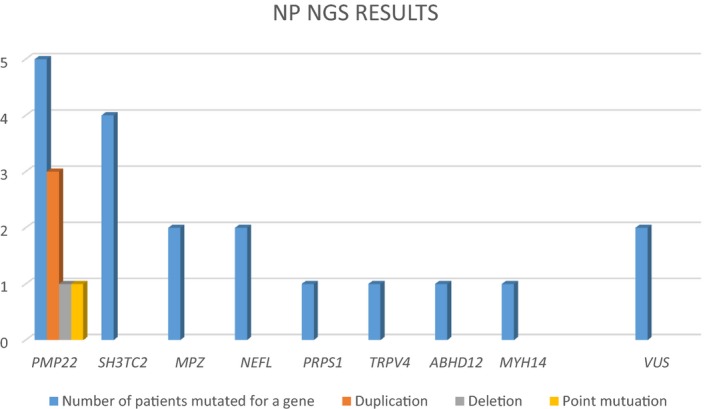
Representation of IPN NGS results of our series

By screening the 36 genes known to be involved in both IPN and HL, pathogenic variants were identified in 16 patients out of 27 (59.26%): *PMP22* (*n* = 5), *SH3TC2* (*n* = 4), *MPZ* (*n* = 2), *NEFL* (*n* = 2), *PRPS1* (*n* = 1), *TRPV4* (*n* = 1), *ABHD12* (*n* = 1) (Figure 1).

The already known variants were: *PMP22* duplication of 1.5Mb in three cases, *PMP22* deletion of 1.4Mb in one case, *PMP22* variant p.(Leu145Argfs*9), *SH3TC2* variants c.2642A>G, p.(Asn881Ser); c.2860C>T, p.(Arg954*); c.3325C>T, p.(Arg1109Ter); c.3596G>A, p.(Trp1199*), *NEFL* variant c.293A>C, p.(Asn98Ser) and *TRPV4* variant c.694C>T, p.(Arg232Cys).

As a consequence, seven novel variants, that could be classified as pathogenic or probably pathogenic, were discovered in five different genes *PRPS1, MPZ, SH3TC2, NEFL* and *ABHD12.* These variants were all absent from the different databases, and in silico* studies* were in favour of pathogenic variants (Tables 3 and 4).

**Figure 2:**
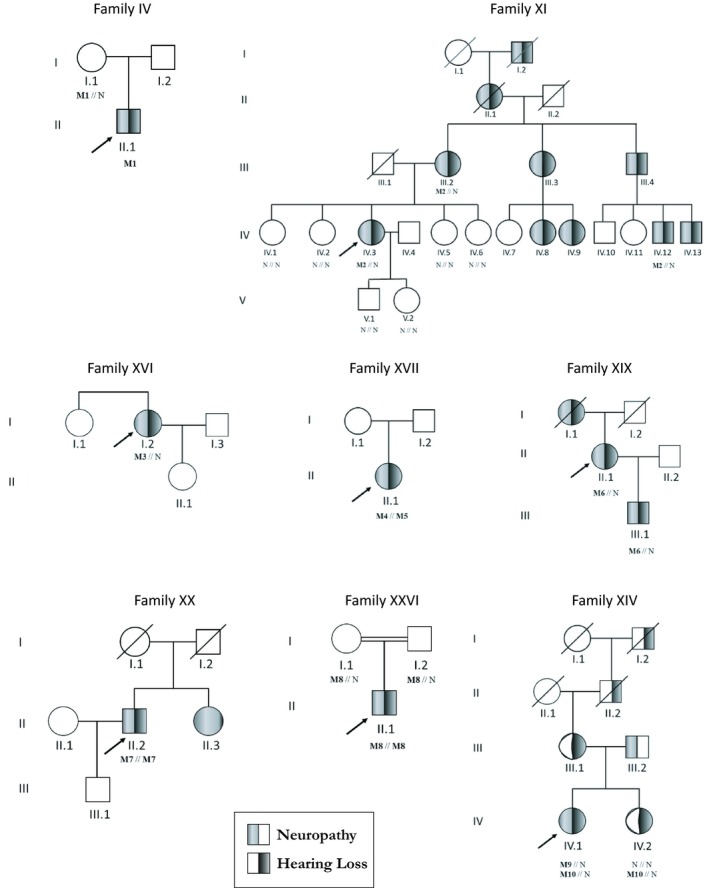
Pedigrees and associated variants ‐ N: Normal allele; M1: c.202A>T, p.(Met68Leu) in *PRPS1*; M2: c.437T>C, p.(Val146Ala) in *MPZ*; M3: c.418T>C, p.(Ser140Pro) in* MPZ*; M4: c.2860C>T, p.(Arg954Ter) in *SH3TC2*; M5: c.3377T>C, p.(Leu1126Pro) in *SH3TC2*; M6: c.269A>G, p.(Glu90Gly) in *NEFL*; M7: c.3617C>A, p.(Ala1206Asp) in *SH3TC2*; M8: c.379_385delAACTACTinsGATTCCTTATATACCATTGTAGTCT, p.(Asn127Aspfs*23) in *ABHD12*; M9: c.434delT, p.(Leu145Argfs*9) in *PMP22*; M10: c.336T>C, p.(Ile109Thr) in *COCH*

‐ c.202A>T, p.(Met68Leu) in *PRPS1:* it was found in patient IV, who developed an X‐linked and axonal form of neuropathy. Family segregation was in accordance with a carrier mother (Figure 2).
‐ c.437T>C, p.(Val146Ala) and c.418T>C, p.(Ser140Pro) in *MPZ*: these two variants were present in patients XI and XVI respectively. Autosomal dominant transmission was suspected in one case. Family segregation was concordant as seen on pedigrees in Figure 2.
‐ c.3377T>C, p.(Leu1126Pro) and c.3617C>A, p.(Ala1206Asp) in *SH3TC2*: patient XVII developed an autosomal recessive form of demyelinating neuropathy and was associated with the known variant c.2860C>T, p.(Arg954*). Family segregation could not have been performed because parents’ DNA was not available.
‐ c. 3617C>A, p.(Ala1206Asp) in *SH3TC2*: Patient XX had an autosomal recessive form of demyelinating neuropathy associated with moderate hearing loss. His sister only developed peripheral neuropathy, but her DNA was not available.
‐ ‐c.269A>G, p.(Glu90Gly) in *NEFL:* patient XIX had an autosomal dominant form of axonal neuropathy. Family segregation was concordant in the son presenting this pathogenic variant associated with IPN and hearing loss.
‐ c.379_385delAACTACTinsGATTCCTTATATACCATTGTAGTCTTACTGCTTTTGGTGAACACA, p.(Asn127Aspfs*23) in *ABHD12*: patient XXVI presented that homozygous variant. Family segregation was concordant, each asymptomatic parent presenting the heterozygous pathogenic variant.

**Table 3:** Genotypes of our 27 patients

Patient			First gene identified						Second gene identified					
Reference Family	Patient (gender/age in years)	Form (Fam/spo)/AD, AR or X‐linked	Gene	Mutation type	Zygosity	Nucleotide change	Amino acid change Localization	dbSNP	Gene	Mutation type	Zygosity	Nucleotide change	Amino acid change Localization	dbSNP
I	F, 87	Spo/ NA	***SPTLC1***	deletion	htz	c.‐35delCCGCTTCCTTCCGGAAGGCGGGTCACAAG		NF	/	/	/	/	/	
II	F, 90	Spo/ NA	/	/	/	/	/		/	/	/	/	/	
III	M, 80	Fam/ AD	/	/	/	/	/		/	/	/	/	/	
IV	M, 35	Spo/ NA	***PRPS1***	missense	hemizygous	**c.202A>T**	**p.(Met68Leu)**	NF	/	/	/	/	/	
V	F, 86	Fam/AD	***PMP22***	duplication of 1.5 Mb	htz	/	/		N2G	N2G	N2G	N2G	N2G	
VI	M, 88	Fam/AD	***DNMT1***	missense	htz	c.1250C>T	p.(Ala417Val)	NF	/	/	/	/	/	
VII	F, 60	Fam/AD	***PMP22***	duplication of 1.5 Mb	htz	/	/		N2G	N2G	N2G	N2G	N2G	
VIII	F, 47	Spo/NA	***SH3TC2***	missense	HMZ	c.3325C>T	p.(Arg1109*)	rs80338934	/	/	/	/	/	
IX	F, 47	Fam/AD	/	/	/	/	/		/	/	/	/	/	
X	M, 69	Fam/AD	***PMP22***	duplication of 1.5 Mb	htz	/	/		N2G	N2G	N2G	N2G	N2G	
X1	F, 68	Fam/AD	***MPZ***	missense	htz	**c.437T>C**	**p.(Val146Ala)**	NF	N2G	N2G	N2G	N2G	N2G	
XII	M, 33	Fam/AD	***PMP22***	deletion of 1.4 Mb	htz	/	/		***MYH14***	htz	missense	c.1067C>T	p.(Thr356Met)	rs151082668
XIII	F, 34	Spo/ NA	/	/	/	/	/		/	/	/	/	/	
XIV	F, 68	Fam/AD	***PMP22***	deletion	htz	c.434delT	p.(Leu145Argfs*9)	rs863225029	***COCH***	htz	missense	c.326T>C	p.(Ile109Thr)	rs1219089
XV	M, 29	Spo/NA	/	/	/	/	/		/	/	/	/	/	
XVI	F, 75	Spo/NA	***MPZ***	missense	htz	**c.418T>C**	**p.(Ser140Pro)**	NF	N2G	N2G	N2G	N2G	N2G	
XVII	♀, 68	Spo/NA	***SH3TC2***	missense x2	htz x2	c.2860C>T + **c.3377T>C**	p.(Arg954Ter) + **p.(Leu1126Pro)**	rs80338933 + NF	/	/	/	/	/	
XVIII	♂, 68	Fam/AD	***TRPV4***	missense	htz	c.694C>T	p.(Arg232Cys)	rs387906904	/	/	/	/	/	
XIX	♀, 69	Fam/AD	***NEFL***	missense	htz	**c.269A>G**	**p.(Glu90Gly)**	NF	N2G	N2G	N2G	N2G	N2G	
XX	♂, 83	Fam/AR	***SH3TC2***	missense	HMZ	**c.3617C>A**	**p.(Ala1206Asp)**	NF	/	/	/	/	/	
XXI	♀, 10	Fam/AD	***NEFL***	missense	htz	c.293A>G	p.(Asn98Ser)	rs58982919	/	/	/	/	/	
XXII	♀, 80	Spo/ NA	/	/	/	/	/		/	/	/	/	/	
XXIII	♂, 19	Spo/ NA	/	/	/	/	/		/	/	/	/	/	
XXIV	♀, 71	Spo/ NA	***SH3TC2***	missense x2	htz x2	c.2642A>G + c.3596G>A	p.(Asn881Ser) + p.(Trp1199*)	rs80338930 + rs761972717	/	/	/	/	/	
XXV	♂, 78	Fam/AD	/	/	/	/	/		/	/	/	/	/	
XXVI	♂, 38	Spo/ NA	***ABHD12***	deletion‐insertion	HMZ	**c.379_385delAACTACTinsGATTCCTTATATACCATTGTAGTCTTACTGCTTTTGGTGAACACA**	p.(Asn127Aspfs*23)	NF	/	/	/	/	/	
XXVII	♂, 61	Spo/ NA	/	/	/	/	/		/	/	/	/	/	

Abbreviations: hmz, homozygous; htz, heterozygous; N2G, no second Gene; NA, not available; NF, not found.

**Table 4:** In silico studies of the seven novel pathogenic variants found with IPN‐NGS

Genes	Mutations	Amino Acid change	Polyphen	SIFT	Mutation Taster	UMD‐Predictor	ExAC
***ABHD12***	**c.379_385delAACTACTinsGATTCCTTATATACCATTGTAGTCTTACTGCTTTTGGTGAACACA**	p.(Asn127Aspfs*23)	/	/	/	/	Not found
***MPZ***	**c.418T>C**	**p.(Ser140Pro)**	0.903 possibly damaging	0.03 deleterious	deleterious	87 pathogenic	Not found
	**c.437T>C**	**p.(Val146Ala)**	0.767 possibly damaging	0.01 deleterious	deleterious	75 pathogenic	Not found
***NEFL***	**c.269A>G**	**p.(Glu90Gly)**	0.999 probably damaging	0 deleterious	/	/	Not found
***PRPS1***	**c.202A>T**	**p.(Met68Leu)**	0.208 benign	0.06 tolerated	deleterious	72 probably pathogenic	Not found
	**c.3377T>C**	**p.(Leu1126Pro)**	0.992 probably damaging	0 deleterious	deleterious	84 pathogenic	Not found
***SH3TC2***	**c.3617C>A**	**p.(Ala1206Asp)**	0.759 possibly damaging	0 deleterious	deleterious	84 pathogenic	Not found

Another rare heterozygous variant in *MYH14,* c.1067C>T, p.(Thr356Met) was found in patient XII, already as being carrier of a 1.4Mb deletion of *PMP22*. *MYH14* was found once in ExAc. The patient had presented a tomacular neuropathy since the age of 20. Severe auditory neuropathy started at the age of 5. Although family segregation was not possible, this variant seems to be potentially pathogenic.

Two additional variants were classified as variants of unknown significance (VUS): patient I presented a novel variant in *SPTCL1*, c. ‐35delCCGCTTCCTTCCGGAAGGCGGGTCACAAG, located in the promotor that could prevent *SPTLC1* expression. However, segregation analysis was not possible, and we cannot conclude for this patient. For patient VI, we found a variant of uncertain significance: c.1250C>T, p.(Ala417Val) in *DNMT1*. It was not present in ExAc. However, this variant involves a residue which is not well conserved among species, and seems to be likely benign.

No other potential pathogenic variant was identified in any other screened genes.

Analysis at the DFNB1 locus did not reveal any pathogenic variant in the 27 patients.

In addition, NGS of the HL‐gene panel was performed in eight selected patients (Patients V, VII, X, XI, XII, XIV, XVI, XIX) carrying IPN and rearrangements in genes frequently involved (*PMP22, MPZ* and *NEFL*). This revealed a known pathogenic variant in *COCH*, c.326T>C, p.(Ile109Thr) (Bae et al., 2014; Pauw et al., 2007) for patient XIV who had a point deletion in *PMP22* (patient XIV with c.434delT, p.[Leu145Argfs*9]). This female patient developed both demyelinating sensori‐motor neuropathy and progressive severe to profound auditory neuropathy at 50. Balance disorders were also reported. Family segregation was concordant, as her sister presented only hearing loss, associated with the *COCH* pathogenic variant.

### 3.3 Phenotype‐Genotype correlations

Hearing loss was mild or moderate in one case of *PMP22* duplication, in cases of variants in *SH3TC2* (*n* = 2), *NEFL* (*n* = 2), *MPZ* (*n* = 1), *TRPV4* (*n* = 1). By contrast, hearing impairment was profound to severe in one case of 1.4Mb deletion *PMP22*, in cases of variants in *PRPS1* and in *ABHD12*.

Hearing loss could develop simultaneously with neuropathy, in some patients with pathogenic variants in *PRPS1* (*n* = 1), *NEFL* (*n* = 2), *MPZ* (*n* = 1), *TRPV4* (*n* = 1); or at a distance in some cases of variants in *SH3TC2* (*n* = 2) and *ABHD12* (*n* = 1). Hearing loss occurrence varied widely with *PMP22.*

In our series, auditory neuropathy was found in five cases: three cases of *PMP22* (1.4Mb deletion, point pathogenic variant), one case due to *TRPV4* and one case due to *ABHD12*. For patient II, no pathogenic variant was identified. Endocochlear hearing loss was observed in patients with variants in *PRPS1, MPZ, SH3TC2, NEFL* and *PMP22* (duplication).

In case of AR demyelinating IPN, *SH3TC2* seems to be the most frequent cause (*n* = 4). This corresponds to CMT4C or AR‐CMTde‐*SH3TC2 (*Mathis et al., 2015 *)*. The frequent association with deafness and/or scoliosis in CMT4C may be a clue for the diagnosis.

Patients who develop polyneuropathy associated with sensorineural hearing loss and optic atrophy during childhood, with an X‐linked inheritance, such as Patient IV, should be assessed for *PRPS1. PRPS1* pathogenic variants lead to CMTX5, a rare condition with only seven variants already reported. Our variant c.202A>T, p.(Met68Leu) is novel.

Pathogenic variants in *NEFL* responsible for CMT are rare and associated to various phenotypes. However, hearing loss is often linked to neuropathy, up to 64% of cases, especially with the pathogenic variants p.(Glu90Lys) and p.(Asn98Ser) (Likar et al., 2018), as it was the case in our p.(Glu90Gly) pathogenic variant. All these pathogenic variants are located in the head domain or in the two ends of the rod domain.

*ABHD12* pathogenic variants lead to a rare phenotype named PHARR syndrome (MIM612674), which is a neurodegenerative disease including demyelinating Polyneuropathy, Hearing loss, cerebellar Ataxia, Retinis pigmentosa and early‐onset Cataract (PHARR). Patient XVI presented demyelinating Polyneuropathy, Hearing loss with auditory neuropathy, Ataxia and Cataracts.

*TRPV4* is responsible for CMT2C or AD‐dHMN‐*TRPV4*. The phenotype is characterized by association with vocal cord and/or diaphragm paresis, and hearing loss (Dyck et al., 1994; Landoure et al., 2012). Patient XVIII’s phenotype corresponds to that clinical presentation.

*MPZ* variants causing axonal neuropathy are often associated with other features, such as hearing loss, or pupil abnormality. A characteristic audiogram of gentle slope curve towards the high frequencies is seen in patients suffering from CMT and sensorineural hearing loss. This was observed in patient XI.

Auditory neuropathy has been associated to *PMP22* variants. As observed in our study, hearing loss associated to neuropathy due to *PMP22* is very variable.

## 4 DISCUSSION

Through the analysis of the literature, we identified 36 genes that have been described to be involved in both IPN and hearing loss. They were all present in the 92 genes custom panel designed for CMT and associated neuropathies diagnosis. They consist in 16 CMT genes, 4 HMN genes, 2 HSN genes, and 14 other IPN genes, mostly syndromic forms. 1.

In our series of 27 patients suffering from both IPN and hearing loss, a molecular diagnosis was made in 16 patients, thus in approximately 60%. Hearing impairment is probably underdiagnosed among PN population. Those 27 patients have been found thanks to medical records and clinical questionnaires among a French series of 3,412 patients suffering from IPN.

In our study, *SH3TC2* seems to be the most frequent gene involved in autosomal recessive demyelinating IPN, CMT4C or AR‐CMTde‐*SH3TC2*, as among 350 patients tested with IPN NGS, 13 had a pathogenic variants in this gene, and four patients were reported deaf. Hearing loss is the most frequent cranial nerve pathology (Azzedine, LeGuern, & Salih, 2008; Piscosquito et al., 2016; Yger et al., 2012). Scoliosis is present in more than one third of this population (Claramunt et al., 2007). Hearing loss frequency (in the patients from our series with variants in *SH3TC2)* is statistically different from that in the general population, showing that the pathogenic variant in *SH3TC2* is directly responsible for hearing loss. We report two novel variants, c.3377T>C, p.(Leu1126Pro) associated with the already known variant, c.2860C>T, p.(Arg954*) and c.3617C>A, p.(Ala1206Asp).

For *ABHD12*, hearing loss is almost constant and is the first clinical sign, starting in the late teens. It is progressive and varies from moderate to profound. IPN is the most variable symptom.

*PRPS1* is linked to three different phenotypes, always associated with hearing loss: CMTX5, DFNX1 and Arts syndrome. These three clinical presentations tend to overlap (Nishikura et al., 2018). In our series of 350 patients tested with NGS, only one patient was diagnosed with this gene, which is a novel hemizygous variant*,* c.202A>T, p.(Met68Leu). This variant is predicted as pathogenic.

For *NEFL*, hearing loss is associated with IPN in case of the following variants: (p.(Glu90Lys), p.(Asn98Ser), p.(Asn98Thr), p.(Leu268Pro), p.(Cys322_Asn326del), p.(Glu396Lys)) (Abe et al., 2009; Fabrizi et al., 2007; Horga et al., 2017; Silvera et al., 2013; Zuchner et al., 2004); and also with our new pathogenic variant: c.269A>G, p.(Glu90Gly). The seven heterozygous variants, including ours, are located on « hot spots» of the protein and seem directly linked to the hearing loss observed in the patients. A tonal audiogram with a moderate slope on the high frequencies is characteristic of variants p.(Asn98Ser) and p.(Glu90Gly) (Likar et al., 2018). The same audiogram was also observed in patient XIX.

*TRPV4* is responsible for CMT2C or AD‐dHMN‐*TRPV4.* The phenotype is characterized by vocal cord paresis and/or diaphragm paresis, and hearing loss (Dyck et al., 1994; Landoure et al., 2012). Patient XVIII’s phenotype corresponds to this description. In our French series, five patients were detected with a variant in *TRPV4*, but only one of them was referred with diagnosed hearing loss.

Interestingly in our series of 3,412 IPN patients, 60 patients were mutated in *MPZ.* Nevertheless, only two patients were reported with hearing impairment (3.33%). Hearing loss frequency does not seem to be statistically different from that in the general population, suggesting that pathogenic variants in *MPZ* may not be the real cause of hearing loss in these patients who are susceptible to carry additional pathogenic variants in HL genes.

Hearing loss has also been described in association with duplication, deletion or point pathogenic variants of the *PMP22* gene (Luigetti, Zollino, Conti, Romano, & Sabatelli, 2013). PMP22 is a major protein expressed in compact myelin of peripheral nerves as well as cranial nerves. Hearing loss in CMT patients is reported with point pathogenic variants or deletions in the transmembrane domain of PMP22, which is in close proximity to the extracellular component of this protein. It has been suggested that pathogenic variants at this site could cause defective interactions with other proteins in Schwann cells, which may result in hypo‐ or demyelination of the peripheral nerves, including the auditory nerve (Postelmans & Stokroos, 2006). Demyelination of the auditory nerve may also be a plausible mechanism to explain the retrocochlear involvement (Verhagen et al., 2005). In addition, endo and retrocochlear hearing loss has been observed in patient presenting the point variant c.193G>T, p.(Val65Phe) (Postelmans & Stokroos, 2006). However, while *PMP22* duplication is responsible for 60% of CMT1, the AD demyelinating type, only few patients in fact suffer from hearing loss. In our series of 3,412 patients, 784 patients were mutated in *PMP22 (23%)* and we had information on associated hearing loss in only 5 of them (0.05%), presenting duplications (*n* = 3), a large deletion (*n* = 1) or 1 basepair deletion (*n* = 1). This 0.05% proportion is statistically different from the rate in the general population, with 10% of hearing loss. Nevertheless, the IPN and CMT populations are younger. It seems difficult to conclude that variations in *PMP22* could protect from hearing loss. We therefore think it is probably underdiagnosed. The rarity of severe hearing loss in families with *PMP22* pathogenic variants could rather suggest that most *PMP22* pathogenic variants have minimal or no effects on hearing loss occurrence. As a consequence, hearing loss in that population could be due to other genes, as we started to point out for two of our patients, with a pathogenic variant in *COCH* and a suspected one in *MYH14.*

The *COCH* gene is responsible for DFNA9, which consists in post‐lingual progressive hearing loss with vestibular dysfunction, such as Meniere‐like diseases (Manolis et al., 1996). The cochline protein is detected in spindle‐shaped cells located along nerve fibers between the auditory ganglion and the sensory epithelium. Patient XIV presented with progressive severe to profound hearing loss, with desynchronised ABR and absent Acoustic Oto Emission. It is the first case to be reported with IPN so far. She also suffered from balance disorders, which could be due to vestibular dysfunction, as the penetrance is very variable.

Indeed, proprioceptive balance disorders or cerebellar ataxia could be misdiagnosed with vestibular dysfunction. Clinical examination is difficult in those patients suffering from IPN. Therefore, vestibular investigations should be performed in IPN patients suffering from balance disturbances.

*MYH14* can lead to two different conditions: DFNA4 with progressive non syndromic hearing loss starting in the first or second decade of life and leading to severe to profound hearing loss in the fourth decade of life (Firstly described by Mirghomizadeh et al., 2002); or to IPN associated with myopathy, hoarseness, and hearing loss (Choi et al., 2011). This phenotype is only reported in one article. Patient XII had presented severe auditory neuropathy starting at the age of 5 and a tomacular neuropathy since the age of 20. No hoarseness or dysphony was reported. A rare heterozygous variant in *MYH14,* c.1067C>T, p.(Thr356Met), that could potentially explain hearing loss, was found in addition to the 1.4Mb deletion of *PMP22* that explains the IPN. That is in accordance with our hypothesis that *PMP22* is not responsible for hearing loss. *MYH14 c*ould nevertheless be also responsible for IPN. Actually, only one case has been reported with IPN and hearing loss (Choi et al., 2011), and two articles have been published about hearing loss with the same pathogenic variants (Chen et al., 1995; Mirghomizadeh et al., 2002).We can wonder whether a founder effect exists, or if a pathogenic variant in a HL gene close to *MYH14* exists.

Hearing loss is reported regularly in patients suffering from IPN. The pathogenesis of hearing loss in those patients is uncertain, even though the cranial nerves are part of the peripheral nervous system and wrapped by Schwann cells. The hypothesis of retrocochlear dysfunction has been suggested and profound hearing loss is supposed to be due to desynchronization of the cochlear nerve (Anzalone et al., 2018). However, in our study we have shown that hearing impairment could be endocochlear, and not only due to AN, as it was the case for patients XIV. In our series, we noticed that both auditory nerve and cochlear dysfunctions were present, as auditory neuropathy was found in five cases: two cases of *PMP22* (1.4Mb deletion, point pathogenic variant), one case due to *TRPV4* and one case due to *ABHD12* (molecular diagnosis was not made for the last one); and endocochlear hearing loss was observed in patients with variants in *PRPS1, MPZ, SH3TC2, NEFL* and *PMP22* (duplication). That was also demonstrated by Kovach et al. (2002) in a patient presenting a variant in *PMP22.* However in most studies, there is a lack of information concerning testing to clearly distinguish cochlear and neuronal components.

To our knowledge, only three patients suffering from IPN and AN received a cochlear implant (absence of information about CMT type or hearing loss type by Anzalone et al., 2018; auditory neuropathy and absence of variant in *PMP22* or *GJB1* by Goswamy, Bruce, Green, & O’Driscoll, 2012; cochlear and auditory nerve dysfunction with a point pathogenic variant in *PMP22,* c.193G>T, p.(Val65Phe) by Postelmans et al., 2006). Our patient XIV also benefited from this surgery. Cochlear implant can recreate synchronous neuronal activity through the electrostimulation, and thus improves speech understanding. However, progress is slower than in other patients with cochlear implant. Patients describe a final significant benefit.

Moreover, hearing loss can precede, occur at the same time or follow IPN. It can be progressive, and the severity varies from mild to profound. We suggest that audiologic assessment should be made in all patients suffering from IPN, and vice versa, patients suffering from hearing loss should be tested for neuropathic involvement, associated with NGS screening of a large panel including genes involved in syndromic pathologies. Indeed, the delay between the different symptoms can be very long (up to 40 years) and a large NGS screening could help to find the gene involved and so to improve the care of the patient. This is for instance the case of Perrault syndrome type II (MIM# 233400), a rare autosomal recessive condition, characterized by sensorineural hearing loss, gonadic dysgenesis in males and females, and neurological features such as developmental delay or intellectual disability, cerebellar ataxia, motor and sensory peripheral neuropathy. As in patients suffering from IPN and hearing loss, the delay between the onset of the two or more symptoms can be up to 40 years, which leads to underdiagnose this phenotype if the involved genes are not tested in “hearing loss” NGS screening (Lerat et al., 2016).

To our knowledge, it is the first time that hearing loss screening has been carried out by MLPA and Sanger sequencing for *GJB2* and *GJB6*. Analysis at the DFNB1 locus did not reveal any pathogenic variant for all diagnosed and known deaf patients. It is also the first time that HL NGS was tested on this population. However, only eight cases could be tested by HL‐NGS because of availability of the analysis. It would have been more significant to test all the 27 patients suffering from IPN and HL by HL‐NGS so as to give better genotype‐phenotype correlations.

Another possibility to explain both IPN and hearing loss is the presence of modifier genes that will induce that particular phenotype. That is why, for unsolved cases, WES could be very useful to identify new candidate genes for IPN and hearing loss, so as to improve diagnosis and patient care.

In addition, to better understand the physiopathology of neuropathies associated with hearing loss, animal models e.g. in rats and mice, should be developed. Indeed, it could be interesting to perform a biopsy of the auditory nerve and cochlea of wild‐type and affected animals in order to localize accurately, for example by immunochemistry the proteins involved in those two features. However, murine phenotype might be different as the organization differs.

## 5 CONCLUSION

Through an NGS strategy, we have been able to establish a molecular diagnosis in almost 60% of the cases presenting IPN associated with HL. As a consequence, a precise description of the phenotype can help molecular investigations. *PMP22,* and in a lesser proportion *MPZ,* involvement is not enough to explain hearing loss in patients suffering from hereditary peripheral neuropathy. Hearing loss can be due to cochlear impairment and/or auditory nerve dysfunction. As HL is certainly underdiagnosed in IPN patients, we suggest that audiologic tests should be systematically performed in these patients and their DNA should be screened with large NGS panels. This would enhance the diagnosis, help to better understand the physiopahology of IPN + HL and eventually improve patient's care.

## CONFLICT OF INTEREST

None.
