# Supplementary material for: Hearing loss in inherited peripheral neuropathies: Molecular diagnosis by NGS in a French series
Source: Mol Genet Genomic Med. 2019 Aug 8;7(9):e839. doi: 10.1002/mgg3.839 (PMC6732311; doi:10.1002/mgg3.839)
Supplement: Supplementary file 1 [file MGG3-7-e839-s001.docx]

**SUPPLEMENTARY DATA**

*Supp Table 1: 92-gene panel used for NGS. It includes the 44 known CMT genes, 27 genes involved in HSN (Hereditary Sensitive Neuropathy) and HMN (Hereditary Motor Neuropathy) and 21 other genes of interest involved in neuropathies of differential diagnoses.*

*Mean depth have been calculated thanks to three matrices of 16 patients, such as 48 patients in total.*

| GENE | CMT1D | CMT2D | CMT1R | CMT2R | HMN D | HMNR | HSN D | HSN R | Other | Coverage | Mean  Depth |
| --- | --- | --- | --- | --- | --- | --- | --- | --- | --- | --- | --- |
| *AARS* |  | X |  |  |  |  |  |  |  | 100 | 1929 |
| *ABHD12* |  |  |  |  |  |  |  |  | R | 91.92 | 1600 |
| *AIFM1* |  |  |  | X |  |  |  |  |  | 100 | 1157 |
| *ARHGEF10* |  |  |  |  |  |  |  |  | D | 100 | 1719 |
| *ATL1* |  |  |  |  |  |  | X |  |  | 100 | 1349 |
| *ATL3* |  |  |  |  |  |  | X |  |  | 100 | 1215 |
| *ATP7A* |  |  |  |  |  | X |  |  |  | 100 | 927 |
| *BICD2* |  |  |  |  | X |  |  |  |  | 96.81 | 2449 |
| *BSCL2* |  |  |  |  | X |  |  |  |  | 100 | 1637 |
| *CCT5* |  |  |  |  |  |  |  | X |  | 100 | 1939 |
| *CTDP1* |  |  |  |  |  |  |  | X |  | 92.02 | 1706 |
| *C12ORF65* |  |  |  |  |  |  |  |  | R | 100 | 1703 |
| *DCAF8* |  |  |  |  |  |  |  |  | D | 100 | 1983 |
| *DCTN1* |  |  |  |  | X |  |  |  |  | 100 | 2148 |
| *DHTKD1* |  | X |  |  |  |  |  |  |  | 99.03 | 1763 |
| *DNAJB2* |  |  |  | X |  |  |  |  |  | 100 | 1977 |
| *DNM2* | X | X |  |  |  |  |  |  |  | 99.15 | 1960 |
| *DNMT1* |  |  |  |  |  |  | X |  |  | 99.98 | 1791 |
| *DST* |  |  |  |  |  |  |  | X |  | 100 | 1376 |
| *DYNC1H1* |  | X |  |  |  |  |  |  |  | 99.87 | 1826 |
| *EGR2* | X |  | X |  |  |  |  |  |  | 100 | 2115 |
| *FAM134B* |  |  |  |  |  |  |  | X |  | 81.43 | 1488 |
| *FBLN5* |  |  |  |  |  |  |  |  | D/R | 100 | 2258 |
| *FBXO38* |  |  |  |  | X |  |  |  |  | 100 | 1426 |
| *FGD4* |  |  | X |  |  |  |  |  |  | 99.5 | 1420 |
| *FIG4* |  |  | X |  | X |  |  |  |  | 100 | 1483 |
| *GAN* |  |  |  |  |  |  |  |  | R | 95.43 | 1808 |
| *GARS* |  | X |  |  | X |  |  |  |  | 100 | 1225 |
| *GDAP1* |  | X | X | X |  |  |  |  |  | 100 | 1256 |
| *GJB1* | X | X |  |  |  |  |  |  |  | 100 | 1547 |
| *GJB3* |  |  |  |  |  |  |  |  | D/R | 100 | 1719 |
| *GNB4* | X | X |  |  |  |  |  |  |  | 100 | 1106 |
| *HARS* |  | X |  |  |  |  |  |  |  | 100 | 1960 |
| *HINT1* |  |  |  |  |  |  |  |  | R | 100 | 1621 |
| *HK1* |  |  | X |  |  |  |  |  |  | 100 | 2220 |
| *HSPB1* |  | X |  |  | X |  |  |  |  | 100 | 1176 |
| *HSPB3* |  |  |  |  | X |  |  |  |  | 100 | 1476 |
| *HSPB8* |  | X |  |  | X |  |  |  |  | 100 | 2244 |
| *IFRD1* |  |  |  |  |  |  |  |  | X | 100 | 1179 |
| *IGHMBP2* |  |  |  | X |  | X |  |  |  | 100 | 1853 |
| *IKBKAP* |  |  |  |  |  |  |  | X |  | 100 | 1385 |
| *INF2* | X | X |  |  |  |  |  |  |  | 98.39 | 1875 |
| *KARS* |  |  | X | X |  |  |  |  |  | 100 | 1503 |
| *KIF1A* |  |  |  |  |  |  |  | X |  | 99.46 | 1710 |
| *KIF1B* |  | X |  |  |  |  |  |  |  | 100 | 1224 |
| *KIF5A* |  |  |  |  |  |  |  |  | D | 100 | 1849 |
| *LITAF* | X |  |  |  |  |  |  |  |  | 93.06 | 2349 |
| *LMNA* |  |  |  | X |  |  |  |  |  | 100 | 1788 |
| *LRSAM1* |  | X |  |  |  |  |  |  |  | 99.17 | 1702 |
| *MARS* |  | X |  |  |  |  |  |  |  | 100 | 1660 |
| *MED25* |  |  |  | X |  |  |  |  |  | 98.58 | 1338 |
| *MFN2* |  | X |  |  |  |  |  |  |  | 100 | 2140 |
| *MPV17* |  |  |  |  |  |  |  |  | R | 100 | 1635 |
| *MPZ* | X | X | X |  |  |  |  |  |  | 100 | 1671 |
| *MTMR2* |  |  | X |  |  |  |  |  |  | 100 | 1083 |
| *NDRG1* |  |  | X |  |  |  |  |  |  | 100 | 1402 |
| *NEFL* | X | X |  |  |  |  |  |  |  | 100 | 1476 |
| *NGF* |  |  |  |  |  |  |  | X |  | 100 | 2284 |
| *NTRK1* |  |  |  |  |  |  |  | X |  | 99.76 | 1640 |
| *PDK3* |  | X |  |  |  |  |  |  |  | 100 | 766 |
| *PLEKHG5* |  |  | X | X |  | X |  |  |  | 96.63 | 1538 |
| *PMP22* | X |  |  |  |  |  |  |  |  | 100 | 2227 |
| *POLG* |  |  |  |  |  |  |  |  | D/R | 99.76 | 1922 |
| *PRPS1* |  |  |  | X |  |  |  |  |  | 100 | 851 |
| *PRX* |  |  | X |  |  |  |  |  |  | 99.62 | 1620 |
| *RAB7A* |  | X |  |  |  |  |  |  |  | 100 | 1270 |
| *REEP1* |  |  |  |  | X |  |  |  |  | 100 | 1028 |
| *SBF1* |  |  | X |  |  |  |  |  |  | 97.17 | 1680 |
| *SBF2* |  |  | X |  |  |  |  |  |  | 99.83 | 1193 |
| *SCN9A* |  |  |  |  |  |  |  | X |  | 100 | 971 |
| *SCN10A* |  |  |  |  |  |  | X |  |  | 100 | 1695 |
| *SCN11A* |  |  |  |  |  |  | X |  |  | 100 | 1285 |
| *SEPT9* |  |  |  |  |  |  |  |  | *D* | 99.72 | 1696 |
| *SETX* |  |  |  |  | X |  |  |  |  | 99.81 | 1104 |
| *SH3TC2* |  |  | X |  |  |  |  |  |  | 100 | 1546 |
| *SLC12A6* |  |  |  |  |  |  |  |  | R | 100 | 1291 |
| *SLC5A7* |  |  |  |  | X |  |  |  |  | 100 | 1339 |
| *SMAD3* |  |  |  |  |  |  |  |  | D | 100 | 1292 |
| *SOX10* |  |  |  |  |  |  |  |  | D | 99.52 | 1151 |
| *SPTLC1* |  |  |  |  |  |  | X |  |  | 100 | 1102 |
| *SPTLC2* |  |  |  |  |  |  | X |  |  | 98.81 | 1142 |
| *SURF1* |  |  | X |  |  |  |  |  |  | 90.21 | 1759 |
| *TFG* |  |  |  |  |  |  |  |  | D | 100 | 1245 |
| *TRIM2* |  |  |  | X |  |  |  |  |  | 100 | 1424 |
| *TRPV4* |  | X |  |  | X |  |  |  |  | 100 | 1479 |
| *TTR* |  |  |  |  |  |  |  |  | D | 100 | 1284 |
| *TUBB3* |  |  |  |  |  |  |  |  | D | 76.27 | 1874 |
| *UBQLN2* |  |  |  |  | X |  |  |  |  | 100 | 1145 |
| *VAPB* |  |  |  |  |  |  |  |  | D | 98.71 | 674 |
| *VCP* |  |  |  |  |  |  |  |  | D | 100 | 1220 |
| *WNK1* |  |  |  |  |  |  |  | X |  | 99.58 | 1413 |
| *YARS* | X | X |  |  |  |  |  |  |  | 100 | 1544 |
