# Supplementary material for: Hearing loss in inherited peripheral neuropathies: Molecular diagnosis by NGS in a French series
Source: Mol Genet Genomic Med. 2019 Aug 8;7(9):e839. doi: 10.1002/mgg3.839 (PMC6732311; doi:10.1002/mgg3.839)
Supplement: Supplementary file 2 [file MGG3-7-e839-s002.docx]

***Supp. Table S2:*** *63-gene panel used for Deafness-NGS.*

*Design was performed with NimbleGen SeqCap EZ Choice technology, sequencing performed with Illumina MiniSeq system, and analysis with LocalRunManager (v1.3.1).*

*The mean coverage for the targeted 63 Deafness genes (390kb; 1029 exons) was 97.9% >50X*

*The exons with less than 50X were: E1-CLDN14, E2-TMC1, E20-E28-OTOA, E1-RDX,E1-TBC1D24, E1-SCL26A5, E1-TPRN, E1-KCNQ4, E1-ACTG1, E18-DIAPH1, E1-CRYM, E1-DFNA5, E1-MYH1*4

| *GENE* | Reference sequence |
| --- | --- |
| *ACTG1 6*  *ADCY1 20*  *CABP2 7*  *CCDC50 12*  *CEACAM16 7*  *CLIC5 6*  *CLDN14 3*  *COCH 12*  *CRYM 10*  *DFNA5 10*  *DIABLO 7*  *DIAPH1 28*  *DIAPH3 28*  *DFNB59 7*  *EPS8 21*  *ESRRB 11*  *EYA4 20*  *FOXI1 2*  *GIPC3 6*  *GJB3 2*  *GJB2 2*  *GJB6 3*  *GPSM2 15*  *GRHL2 16*  *GRXCR1 4*  *GRXCR2 3*  *HGF 18*  *ILDR1 8*  *KCNJ10 2*  *KCNQ4 14*  *LHFPL5 4*  *LOXHD1 40*  *LRTOMT 7*  *MARVELD2 7*  *MSRB3 6*  *MIR96 1*  *MYH9 41*  *MYH14 43*  *MYO15A 66*  *MYO3A 35*  *MYO6 35*  *OTOA 28*  *OTOG 55*  *OTOGL 58*  *OTOF 47*  *POU3F4 1*  *POU4F3 2*  *PRPS1 7*  *PTPRQ 50*  *RDX 14*  *SERPINB6 7*  *SLC17A8 12*  *SLC26A4 21*  *SLC26A5 20*  *SMPX 5*  *TBC1D24 8*  *TJP2 23*  *TECTA 23*  *TMC1 24*  *TMIE 4*  *TMPRSS3 13*  *TPRN 4*  *WFS1 8* | NM_001614.3  NM_021116.2  NM_016366.2  NM_178335.2  NM_001039213.3  NM_001114086.1  NM_001146077.1  NM_004086.2  NM_001888.3  NM_004403.2  NM_019887.5  NM_005219.4  NM_001042517.1  NM_001042702.3  NM_004447.5  NM_004452.3  NM_004100.4  NM_012188.4  NM_133261.2  NM_024009.2  NM_004004.5  NM_006783.4  NM_013296.4  NM_024915.3  NM_001080476.2  NM_001080516.1  NM_000601.4  NM_001199799.1  NM_002241.4  NM_004700.3  NM_182548.3  NM_144612.6  NM_001145308.4  NM_001038603.2  NM_198080.3  NM_029512.1  NM_002473.4  NM_001145809.1  NM_016239.3  NM_017433.4  NM_004999.3  NM_144672.3  NM_001277269.1  NM_173591.3  NM_194248.2  NM_000307.4  NM_002700.2  NM_002764.3  NM_001145026.1  NM_002906.3  NM_001195291.2  NM_139319.2  NM_000441.1  NM_198999.2  NM_014332.2  NM_001199107.1  NM_004817.3  NM_005422.2  NM_138691.2  NM_147196.2  NM_024022.2  NM_001128228.2  NM_006005.3 |
